# Supplementary material for: HDHL-INTIMIC: A European Knowledge Platform on Food, Diet, Intestinal Microbiomics, and Human Health
Source: Nutrients. 2022 Apr 29;14(9):1881. doi: 10.3390/nu14091881 (PMC9100002; doi:10.3390/nu14091881)
Supplement: Supplementary file 1 [file nutrients-14-01881-s001.zip › nutrients-1680302-supplementary.pdf]

Organisation name	Acronym organisation
Amedes genetics GmbH	AME
Ben-Gurion University of the Negev	BGU
Leibniz Institute for Prevention Research and Epidemiology - BIPS	BIPS
Bonn University	BU
Department of Endocrinology, Diabetology and Clinical Nutrition, University of Kiel	CAU
Chalmers University, Division Food and Nutrition Science, Department of Biology and Biological Engineering	CHALMERS
Centre d'Immunologie et des Maladies Infectieuses	CIMI
	CNR-IBBA
Consiglio Nazionale delle Ricerche- Istituto di Fisiologia Clinica	CNR-IFC
Research Centre for Food and Nutrition, CREA (Council for Agricultural Research and Economics)	CREA
Human Nutrition Research Center	CRNH-RA
Helmholtz Zentrum München	HMGU
Hospital Regional Universitario de Málaga-Instituto de Investigación Biomédica de Málaga	IBIMA-FIMABIS
Flanders Research Institute for Agriculture, Fisheries and Food	ILVO
Institute of Network Biology, Helmholtz Zentrum München	INET
INRA Unité de Nutrition Humaine	INRA-UNH
INSERM-University of Rouen	INSERM-UR
Instituto de Productos Lácteos de Asturias	IPLA-CSIC
Istituto Superiore di Sanità	ISS
Max Delbrueck Center for Molecular Medicine	MDC
INRA/ University of Clermont Auvergne	MEDIS
Institute for Laboratory Animal Science, Hannover Medical School	MHH
Institute of Clinical Microbiology and Hygiene, University Hospital Regensburg	MHR
Migal - Galilee Research Institute	MIGAL
Max Rubner-Institut	MRI
Medical University of Graz	MUG
Medical University of Vienna	MUW
The Open University of Israel	OPENU
Sigmund Freud University Vienna	SFU
Netherlands Organisation for applied scientific research	TNO
University of Barcelona, Nutrition & Food Science Department	UB
Gothenburg University	UGOT
University of Gothenburg	UGOT
University of Gothenburg	UGOT
Institute of Animal Science, University of Hohenheim,	UHO
University Clinic RWTH Aachen, Medical Clinic III	UH-RWTH
NUTRIM School of Nutrition and Translational Research in Metabolism, Maastricht University	UM
University Medical Center Groningen	UMCG
University of Milan, Department of Food, Environmental and Nutritional Sciences	UMIL
University of Milano, Department of Pharmacological and Biomolecular Sciences	UMIL
University of Bari Aldo Moro	UNIBA
Alma Mater Studiorum University of Bologna	UNIBO
Faculty of Science and Technology, Free University of Bolzano	UNIBZ
University of Florence, Department of Biology	UNIFI-BIO
University of Florence, NEUROFARBA Department	UNIFI-NEUROFARBA
University of Graz	UNIGRAZ
Unilever Research & Development	UNILEVER
University of Naples Federico II	UNINA
Federico II University Dept Clinical Medicine and Surgery	UNINA2
University of Turin	UNITO
University of Vienna	UNIVIE
University of Veterinary Medicine Vienna, Institute for Milk Hygiene, Milk Technology and Food Science	VMU
Wageningen University and Research	WUR
